# Routine jejunostomy feeding after esophagectomy reduces early weight loss: a cohort study

**DOI:** 10.1007/s00423-025-03944-w

**Published:** 2026-01-09

**Authors:** Jessica Yan-Seen Ng, Catherine Jenn Yi Cheang, Alexander W. Phillips

**Affiliations:** 1 The Northern Oesophago-Gastric Unit, Royal Victoria Infirmary, Newcastle upon Tyne, UK; 2 Upper GI Surgery Department, 199 Ipswich Rd, Woolloongabba, QLD 4102 Australia; 3 School of Medical Education, Newcastle University, Newcastle upon Tyne, UK

**Keywords:** Esophagectomy, Nutrition, Enteral nutrition, Esophageal cancer, Jejunostomy

## Abstract

**Purpose:**

Jejunostomy feeding tubes are commonly used during esophagectomy to provide postoperative nutritional support, but their impact on clinical outcomes remains uncertain.

**Methods:**

This retrospective study included 507 patients who underwent esophagectomy for esophageal cancer at the Northern Oesophagogastric Unit between 2012 and 2014 and 2016–2019. Routine postoperative jejunostomy feeding was introduced in 2015. Outcomes measured were percentage weight change at 2, 6, 12, and 24 weeks postoperatively, length of hospital stay, weight loss in patients with major complications (Clavien-Dindo > 3), and long-term survival.

**Results:**

Of the 507 patients, 290 received routine jejunostomy feeding and 217 did not. Jejunostomy-fed patients experienced significantly less weight loss at all measured time points, with weight loss at 2 weeks of 3.22% versus 7.24% (*p* < 0.001), at 6 weeks of 3.28% versus 8.30% (*p* < 0.001), at 3 months of 5.49% versus 10.38% (*p* < 0.001), and at 6 months of 6.89% versus 11.46% (*p* = 0.006). The greatest benefit was observed in patients receiving neoadjuvant chemotherapy followed by surgery, with the most pronounced difference at 3 months (5.23% vs. 11.63%). Median hospital stay was shorter in the jejunostomy group (11 versus 15 days), and long-term survival tended to be higher among jejunostomy-fed patients, although this did not reach statistical significance, although there was no significant difference in weight loss among patients with major complications.

**Conclusion:**

Routine postoperative jejunostomy feeding significantly reduces early postoperative weight loss after esophagectomy, particularly in patients receiving neoadjuvant chemotherapy. These benefits diminish by six months. Jejunostomy feeding may also shorten hospital stay and could be associated with improved long-term survival.

## Introduction

Esophageal cancer treatment primarily involves surgical resection, often combined with neoadjuvant therapy, particularly in advanced stages. During esophagectomy, jejunostomy feeding tubes are frequently inserted to provide early postoperative nutritional support. While these tubes are intended to maintain nutrition and mitigate the effects of complications, their use may also be associated with increased morbidity.

Evidence suggests that early nutritional support through jejunostomy feeding can enhance recovery following major abdominal surgery [1]. Despite tailored dietary plans, patients commonly experience poor nutrition postoperatively, highlighting the need for additional supplementary feeding methods [2]. Home enteral nutrition via jejunostomy has been shown to enhance nutritional status, reduce fatigue, and lower the risk of postoperative pneumonia, contributing to overall improvements in quality of life for patients recovering from esophagectomy [3]. Many patients present with significant weight loss and malnutrition both at diagnosis and after treatment, and maintaining adequate nutrition is essential in reducing the risk of postoperative complications, lowering reoperation rates, and improving long-term survival.

Malnutrition negatively impacts treatment outcomes for patients with esophageal cancer after esophagectomy and is often worsened by chemotherapy and radiotherapy [4]. Consequently, strategies to ensure adequate nutritional intake are crucial. Jejunostomy feeding can provide a reliable means of nutrition, particularly for patients who develop postoperative complications, thereby helping to reduce the risk of complications and promote recovery [5].

This study evaluates the impact of routine postoperative jejunostomy feeding on weight loss in patients who have undergone esophagectomy, aiming to determine its effectiveness in mitigating the nutritional challenges faced during recovery.

## Method and materials

### Patient population

A retrospective review of patients who underwent esophagectomy for esophageal cancer between January 2012 to December 2014 and January 2016 to December 2019 at the Northern Oesophagogastric Unit, Newcastle Upon Tyne was performed. The year 2015 was excluded as a transition period when routine jejunostomy feeding was being introduced, to avoid bias and ensure clear comparison between cohorts. Routine post-operative feeding via jejunostomy, including overnight feeding upon discharge, was introduced in 2015; since then, all patients undergoing esophagectomy have received a jejunostomy feeding tube as part of standard care. All patients had a feeding jejunostomy placed prior to 2015; however, these tubes were not routinely used for discharge feeding but were intended as a precautionary measure in case patients experienced difficulties during adjuvant chemotherapy. We sought to investigate long term outcomes through review of electronic medical records and an existing patient database.

### Staging and treatment

Initial staging comprised of endoscopy with biopsy, endoscopic ultrasonography, and positron emission tomography scans. Neoadjuvant therapy followed by esophagectomy was indicated for patients with histologically proven, advanced, resectable malignancy without distant metastases (cT1, N1-3, M0 or cT2-3, N0-3, M0) and/or those with tumors of questionable resectability (cT4). The types of esophagectomy performed during the study periods (2012–2014 and 2016–2019) included two-stage (Ivor Lewis) and three-stage (McKeown) procedures, both with stapled anastomoses. Feeding jejunostomy was placed approximately 20–30 cm distal to the duodenojejunal flexure using a Witzel tunnel technique. Omentopexy was performed to secure the jejunostomy to the abdominal [6]. There were no significant differences in patient selection criteria or variations in nutritional support protocols beyond the use of jejunostomy feeding during these time periods. Patients with metastatic disease, non-resectable tumors at exploratory surgery were excluded. Patients with T2N0 disease or earlier received surgery alone unless otherwise indicated. Patients with early disease amenable to endoscopic mucosal resection were excluded.

### Neoadjuvant therapies and operative treatment

Multiple neoadjuvant regimens were employed based on the standard of care and available clinical trials at the time of treatment. The majority of patients received epirubicin, cisplatin, and either fluorouracil or capecitabine (ECF/ECX - MAGIC regimen). FLOT (Fluorouracil, Leucovorin, Oxaliplatin, and Docetaxel) was increasingly used as a neoadjuvant regimen after 2017. Esophagectomy was performed within 8 weeks after completion of neoadjuvant therapy using a conventional approach.

### Outcomes

Primary outcomes measured included percentage weight change at 2 weeks, 6 weeks, 3 months, and 6 months compared to initial weight at diagnosis, with subgroup analyses based on different types of multimodal cancer therapy used. Secondary outcomes investigated included length of hospital stay, weight loss in patients with complications greater than Clavien-Dindo 3, and long-term survival duration.

### Follow-up and definition of recurrence

All patients were followed up at outpatient clinics at 3- to 6-month intervals for the first 2 years, then every 6 months to annually up until 5 years. Recurrence was diagnosed based on clinical suspicion and confirmed with either CT imaging or endoscopy.

### Statistical analysis

Continuous variables were compared using t-tests for equality of means. Categorical variables were compared using Fisher’s exact test. A P-value of < 0.05 was considered statistically significant. Analyses were performed using SPSS version 26.0 (IBM Corp., Armonk, NY).

## Results

### Patient characteristics

This study included 507 patients who underwent esophagectomy for cancer between January 2012 and December 2014, and January 2016 and December 2019 at the Northern Oesophagogastric Unit, Newcastle Upon Tyne. Of these, 290 patients received routine postoperative feeding through a jejunostomy, which was introduced in 2015, while the remaining 217 patients did not receive routine jejunostomy feeding. Patient characteristics are summarized in Table 1.

**Table 1:** Patient demographics

	No Jejunostomy (*n*) = 217	With jejunostomy (*n*) = 290	Significance (*p*)
Age at surgery	64.6 ± 9.8	65.6 ± 8.5	0.247
Male Gender (%)	74.2 (161/217)	76.2 (221/290)	0.603 (Pearson’s chi)
Ivor Lewis (%)	96.3 (209/217)	91.7 (266/290)	
McKeown (%)	3.7 (8/217)	8.3 (24/290)	
**Co-morbidities (n)**			
Cardiac disease	92	137	0.281(Fishers)
Respiratory disease	39	47	0.633(Fishers)
Diabetes Mellitus	22	46	0.066(Fishers)
Renal disease	12	12	0.528 (Fishers)
Liver disease	2	2	1.000 (Fishers)
**Treatment combination (n)**			
NAC* + Surgery + AC**	33	53	0.166 (Pearson Chi)
NAC* + Surgery	110	118	
Surgery + AC**	2	4	
Surgery only	72	115	
**Histology (n)**			
Adenocarcinoma	160	214	0.148 (Pearson chi)
Adenosquamous	3	3	
Squamous cell carcinoma	44	67	
High grade dysplasia	7	5	
Others	3	0	
**Site of cancer (n)**			
Upper esophagus	2	6	0.043 (Pearson chi)
Middle esophagus	28	57	
Lower esophagus	150	166	
Gastro-esophageal junction	36	59	
**T stage (n)**			
T0	6	25	0.006 (Pearson chi)
T1	44	81	
T2	36	42	
T3	110	128	
T4	16	11	
**N Stage (n)**			
N0	103	152	0.611 (Pearson chi)
N1	53	67	
N2	38	40	
N3	22	30	

*NAC Neoadjuvant chemotherapy, **AC Adjuvant chemotherapy

### Weight change post-surgery

Overall, patients who received jejunostomy feeding consistently demonstrated less weight loss compared to those without jejunostomy feeding across all time points and treatment groups. (Table 2) This difference was most pronounced in the first 6 months postoperatively (Tables 3 and 4).

**Table 2:** (Results): primary outcomes: mean weight loss in kilograms

	Mean weight loss in kg (%) ± SEM* (*n*)		Significance (*P*-value)
	No jejunostomy feeding	With jejunostomy feeding	
All Patients			
Week 2 (kg)	5.50 (7.24%) ± 0.28 (193)	2.92 (3.22%) ± 0.22 (256)	< 0.001
Week 6 (kg)	6.37 (8.30%) ± 0.33 (214)	3.39 (3.28%) ± 0.25 (258)	< 0.001
3 Months (kg)	7.96 (10.38%) ± 0.47 (172)	4.81 (5.49%) ± 0.30 (258)	< 0.001
6 Months (kg)	9.28 (11.46%) ± 0.49 (214)	7.25 (6.89%) ± 0.53 (247)	0.006
NAC** + Surgery + AC***			
Week 2 (kg)	5.55 (7.01%) ± 0.81 (24)	3.05 (3.32%) ± 0.45 (54)	0.005
Week 6 (kg)	6.01 (7.77%) ± 1.02 (26)	3.38 (4.13%) ± 0.57 (54)	0.017
3 Months (kg)	7.08 (8.81%) ± 1.69 (23)	5.37 (5.23%) ± 0.72 (54)	0.272
6 Months (kg)	9.14 (10.86%) ± 1.46 (28)	6.29 (9.26%) ± 0.77 (53)	0.060
Surgery only			
Week 2 (kg)	5.00 (6.90%) ± 0.49 (73)	3.07 (3.60%) ± 0.32 (97)	< 0.001
Week 6 (kg)	5.80 (7.86%) ± 0.50 (77)	3.31 (3.87%) ± 0.37 (98)	< 0.001
3 Months (kg)	6.73 (9.45%) ± 0.62 (61)	5.03 (5.69%) ± 0.44 (98)	0.024
6 Months (kg)	8.72 (11.32%) ± 0.77 (78)	7.00 (7.77%) ± 0.63 (94)	0.084
NAC** + surgery			
Week 2 (kg)	6.02 (7.70%) ± 0.36 (91)	2.70 (3.32%) ± 0.40 (104)	< 0.001
Week 6 (kg)	7.04 (8.88%) ± 0.48 (106)	3.45 (4.13%) ± 0.42 (105)	< 0.001
3 Months (kg)	9.25 (11.63%) ± 0.68 (84)	4.32 (5.23%) ± 0.48 (105)	< 0.001
6 Months (kg)	9.90 (11.81%) ± 0.72 (103)	8.02 (9.26%) ± 1.12 (99)	0.157

*SEM Standard Error of Mean, **NAC Neoadjuvant chemotherapy, ***AC Adjuvant chemotherapy

**Table 3:** Secondary outcome measures

	No Jejunostomy	With jejunostomy	Significance (*p*)
Length of stay (days)	15 ± 11 (182)	11 ± 9 (290)	< 0.05
All complications (including Clavien-Dindo < 3)	66.5% (109/164)	67.9% (230/339)	0.75
Clavien-Dindo classification of complications > 3 (%)	26.1% (49 / 188)	27.5% (128/475)	0.75

**Table 4:** Numbers at risk

Time (months)	Jejunostomy (Number at Risk)	No Jejunostomy (Number at Risk)
0	290	217
12	278	205
24	266	193
36	254	181
48	242	169
60	230	157
72	218	145
84	206	133
96	194	121
108	182	109
120	170	97
140	131	126
160	135	135
180	137	133
200	138	138

For the entire cohort, patients without jejunostomy feeding lost significantly more weight at all time points. At 2 weeks post-surgery, patients without jejunostomy lost 5.50 kg (7.24%) compared to 2.92 (3.22%) kg in those with jejunostomy (*p* < 0.001). This trend continued at 6 weeks (6.37 kg (8.30%) vs. 3.39 kg (3.28%), *p* < 0.001) and 3 months (7.96 kg (10.38%) vs. 4.81 kg (5.49%), *p* < 0.001). By 6 months, although the difference narrowed, it remained statistically significant (9.28 kg (11.46%) vs. 7.25 kg (6.89%), *p* = 0.006).

When analyzing subgroups based on treatment regimens, we observed varying patterns. In the neoadjuvant chemotherapy (NAC) with surgery and adjuvant chemotherapy (AC) group, significant differences in weight loss were observed at 2 weeks (5.55 kg (7.01%) vs. 3.05 kg (3.32%), *p* = 0.005) and 6 weeks (6.01 kg (7.77%) vs. 3.38 kg (4.13%), *p* = 0.017). However, by 3 months and 6 months, the differences were no longer statistically significant.

In the surgery-only group, significant differences in weight loss persisted up to 3 months post-surgery. At 2 weeks, patients without jejunostomy lost 5.00 kg (6.90%) compared to 3.07 kg (3.60%) in those with jejunostomy (*p* < 0.001). This difference remained significant at 6 weeks (5.80 kg (7.86%) vs. 3.31 kg (3.87%), *p* < 0.001) and 3 months (6.73 kg (9.45%) vs. 5.03 kg (5.69%), *p* = 0.024). By 6 months, the difference was no longer statistically significant (8.72 kg (11.32%) vs. 7.00 kg (7.77%), *p* = 0.084).

The NAC and surgery group demonstrated the most pronounced differences in weight loss. At 2 weeks, patients without jejunostomy lost 6.02 kg (7.70%) compared to 2.70 kg (3.32%) in those with jejunostomy (*p* < 0.001). This substantial difference persisted at 6 weeks (7.04 kg (8.88%) vs. 3.45 kg (4.13%), *p* < 0.001) and was most noticeable at 3 months, where patients without jejunostomy lost over twice as much weight (9.25 kg (11.63%) vs. 4.32 kg (5.23%), *p* < 0.001). However, by 6 months, the difference was no longer statistically significant (9.90 kg (11.81%) vs. 8.02 kg (9.26%), *p* = 0.157).

These results suggest that jejunostomy feeding is most effective in reducing weight loss in the early postoperative period, particularly within the first 3 months after surgery. The benefits appear to be most pronounced in patients receiving neoadjuvant chemotherapy. However, the impact of jejunostomy feeding on weight loss seems to diminish over time, with differences becoming non-significant by 6 months in most treatment groups.

A significantly higher proportion of patients in the jejunostomy group received adjuvant treatment compared to those without a jejunostomy (21.2% vs. 12.4%), suggesting that jejunostomy placement may be associated with increased uptake of adjuvant chemotherapy.

### Length of hospital stay

Median length of stay for patients who received jejunostomy feeding was significantly shorter (11 days ± 9), compared to those who did not receive jejunostomy feeding (15 days ± 11), *p* = 0.05.

### Weight loss in patients with complications greater than Clavien-Dindo 3

There was no significant difference in weight loss in patients who experienced severe complications based on the Dindo severity grading system.

### Long-term survival duration

**Fig. 1:**
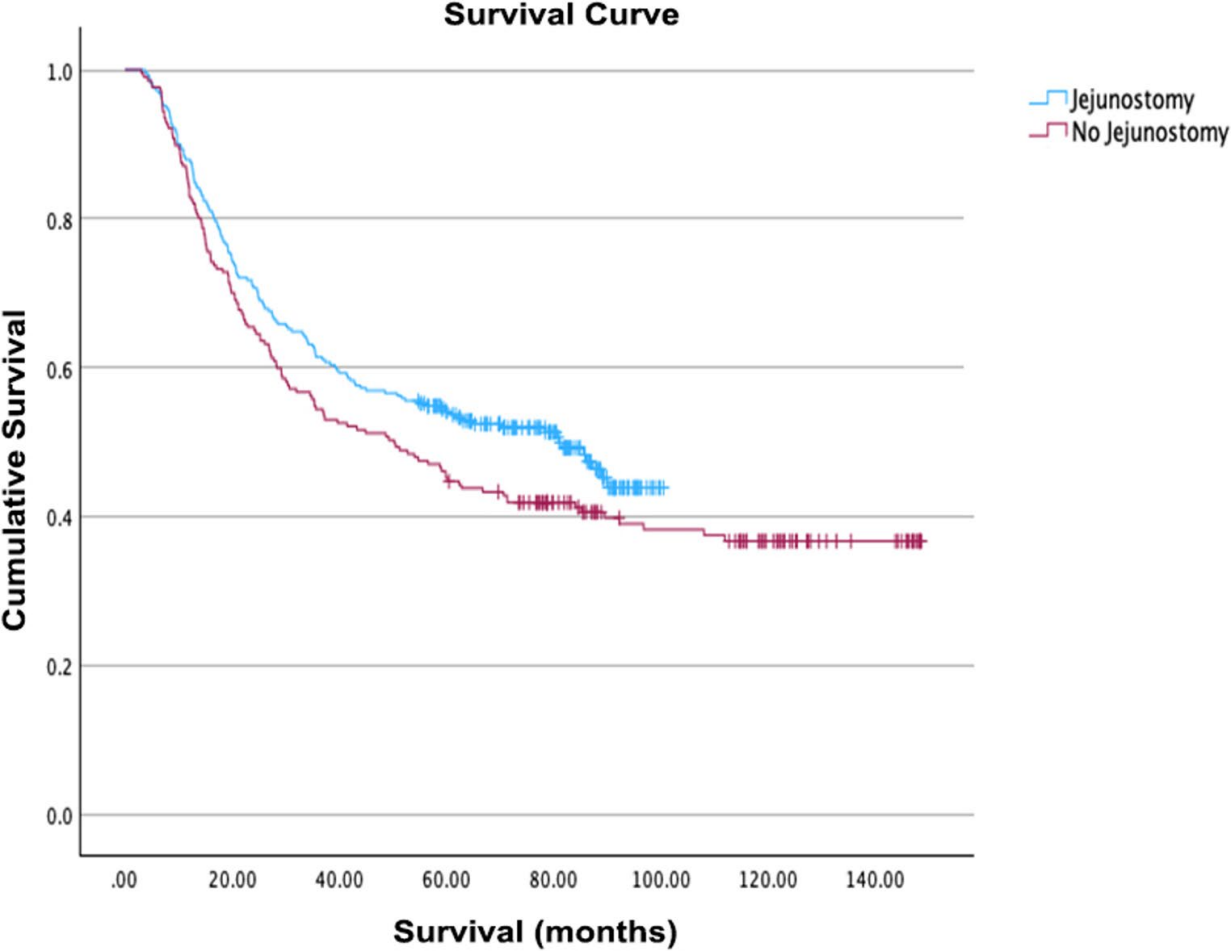
Kaplan Meier survival curve

Long-term survival outcomes after esophagectomy were compared between patients who received routine postoperative jejunostomy feeding and those who did not, using Kaplan-Meier analysis. The survival curves demonstrate a visible separation in favor of the jejunostomy group (Fig. 1). Median survival was higher in the jejunostomy group (81.1 months, 95% CI: 62.4–99.8) compared to the non-jejunostomy group (50.2 months, 95% CI: 34.6–65.8). However, the difference did not reach statistical significance by the log-rank test (χ² = 2.82, *p* = 0.093), indicating that the observed difference may represent a trend rather than a definitive survival benefit within this cohort.

## Discussion

The results of this study show that jejunostomy feeding offers significant benefits in the short term, particularly in reducing weight loss in the early postoperative period. However, the long-term effects of jejunostomy feeding on clinical outcomes such as survival and functional recovery require further investigation. Moreover, additional research is needed to identify patient populations that would benefit most from routine jejunostomy placement, ensuring that its use provides optimal nutritional support while minimizing potential complications.

The use of jejunostomy feeding tubes after esophagectomy remains a subject of ongoing debate. While jejunostomy placement offers a reliable route for postoperative nutritional support, it is not without its complications. Previous studies have documented several jejunostomy-related issues, including bowel obstruction [7, 8] infections at the insertion site, ileus, and frequent emergency room visits [5, 9]. A recent meta-analysis of 12 studies found that routine jejunostomy placement was associated with longer operating times [10, 11] and higher rates of morbidity and wound infection [12]. Furthermore, jejunostomy feeding does not consistently prevent weight loss in the long term, nor does it uniformly improve nutritional status compared to oral feeding [1, 8].

Despite these challenges, evidence suggests that jejunostomy feeding may lower 30-day mortality without significantly influencing other common postoperative complications [11]. Additionally, routine jejunostomy placement can improve short-term nutritional outcomes and may be associated with better long-term survival [10]. However, there remains conflicting evidence, with some studies reporting no significant differences in clinical outcomes such as anastomotic leak rates, protein levels, or the need for further nutritional interventions [13, 14].

Postoperative weight loss following esophagectomy is clinically significant because it is linked to poorer nutritional status, decreased tolerance to adjuvant therapies, and reduced overall survival, especially in patients with advanced disease. Kubo et al. showed that patients who lost 12% or more of their body weight six months after surgery experienced significantly worse survival outcomes, highlighting the importance of strategies to minimize weight loss in this population [15]. Additionally, malabsorption commonly occurs after esophagectomy, further contributing to nutritional deficiencies and weight loss [16]. This underscores the value of enteral feeding via jejunostomy, which may help mitigate malabsorption by ensuring adequate nutrient delivery in the early postoperative period. The percentage of weight loss observed in our study is consistent with the ranges reported in the literature. Prior studies have described a mean weight loss of 11.7% at six months post-esophagectomy, with up to 62.9% of patients experiencing >10% unintentional weight loss, and some subgroups, losing as much as 16.2% of their preoperative weight. Another prospective cohort study reported an average 8% weight loss at six months, with 20% of patients losing more than 15% [17]. In our cohort, patients without jejunostomy feeding lost between 10.38% and 11.81% of their body weight by six months, while those with jejunostomy feeding generally lost less (6.89% to 9.26%). Our findings indicate that patients who received jejunostomy feeding consistently demonstrated less weight loss across all time points, particularly in the early postoperative period (up to 3 months). This effect was especially pronounced in patients undergoing neoadjuvant chemotherapy in combination with surgery, reinforcing the importance of jejunostomy feeding in reducing early postoperative weight loss. Furthermore, routine jejunostomy feeding was associated with a significantly shorter length of hospital stay, although no significant difference was observed in the occurrence of severe complications.

Achieving adequate nutritional status is particularly relevant for patients who require adjuvant chemotherapy following neoadjuvant therapy and esophagectomy, as this approach has been associated with improved survival, even in node-negative and margin-negative disease [18]. Since malnutrition can delay or prevent patients from receiving adjuvant therapy, routine use of jejunostomy feeding may play a crucial role in optimizing postoperative recovery and ensuring patients can proceed with further oncological treatment.

A higher proportion of patients in the jejunostomy group received adjuvant treatment compared to those without a jejunostomy (21.2% vs. 12.4%), suggesting a potential association between jejunostomy placement and greater uptake of adjuvant chemotherapy. While this may reflect an additional benefit beyond nutritional support, the retrospective nature of the study limits interpretation. Confounding factors such as differences in chemotherapy regimens, comorbidities, treatment tolerance, patient preference, social support, health literacy, and time to recovery may have influenced access to or suitability for adjuvant treatment independently of jejunostomy use.

Due to the retrospective design and limitations in data retrieval, a detailed assessment of jejunostomy-related complications was not feasible. A limitation of this study is the introduction of bias due to the simultaneous introduction of routine jejunostomy feeding and the adoption of chemotherapy regimens. Additionally, this study compares two non-concurrent cohorts from different time periods (2012–2014 and 2016–2019), introducing temporal bias that may further confound the observed outcomes. Since the two changes occurred concurrently, it is difficult to entirely separate the effects of the feeding protocol from the potential impact of neoadjuvant chemotherapy on postoperative recovery and nutritional status. The potential confounding effect of neoadjuvant chemotherapy on the observed outcomes as well as evolving surgical techniques should therefore be considered when interpreting the results. Enhanced recovery after surgery protocols were introduced in 2016, and these have been shown to improve both short and long term outcomes. Such changes in discharge criteria, hospital policies, and postoperative care pathways may have independently impacted the outcomes observed and represent potential confounding factors not fully controlled for in this retrospective analysis [19]. Future studies with a more controlled design and clearer distinction between the effects of nutritional support and chemotherapy could provide further insight into the specific role of jejunostomy feeding.

Previous research has emphasized the importance of implementing perioperative pathways to optimize patient recovery and reduce the risk of complications [20]. In this study, we sought to determine whether the routine use of jejunostomy feeding could contribute to reducing the effects of postoperative complications by improving nutritional status and minimizing excessive weight loss. Additionally, it is important to note that we do not have data on functional improvement or quality of life (QoL) in our cohort. Recent research by Jia et al. highlights that esophageal cancer survivors who experience greater weight loss and higher symptom distress tend to have significantly worse QoL, underscoring the clinical importance of minimizing postoperative weight loss to improve patient-centered outcomes [21]. While our findings suggest that early enteral feeding may be a valuable component of perioperative care, we did not observe a significant difference in the incidence of severe complications between patients with or without jejunostomy feeding.

## Conclusion

This study underscores the significant role of routine jejunostomy feeding in reducing short-term weight loss after esophagectomy. Jejunostomy feeding is associated with improved early postoperative nutrition and a reduced length of hospital stay, offering valuable support to patients during their recovery. However, the long-term clinical benefits, including its impact on survival, long-term nutritional status, and functional recovery, remain unclear and warrant further research.

## Data Availability

No datasets were generated or analysed during the current study.

## References

[1] Carroll PA, Yeung JC, Darling GE (2020) Elimination of routine feeding jejunostomy after esophagectomy. Ann Thorac Surg 110:1706–1713. 10.1016/j.athoracsur.2020.04.072 32504612 10.1016/j.athoracsur.2020.04.072

[2] Froghi F, Sanders G, Berrisford R, Wheatley T, Peyser P, Rahamim J, Lewis S (2017) A randomised trial of post-discharge enteral feeding following surgical resection of an upper Gastrointestinal malignancy. Clin Nutr 36:1516–1519. 10.1016/j.clnu.2016.10.022 27842926 10.1016/j.clnu.2016.10.022

[3] Zhang C, Hu LW, Qiang Y, Cong ZZ, Zheng C, Gu WF et al (2022) Home enteral nutrition for patients with esophageal cancer undergoing esophagectomy: A systematic review and meta-analysis. Front Nutr 9:895422. 10.3389/fnut.2022.895422 35967793 10.3389/fnut.2022.895422 PMC9366554

[4] Starr B, Davis S, Ayala-Peacock D, Blackstock WA, Levine EA (2014) Reassessment of the role of enteral tube feedings for patients with esophageal cancer. Am Surg 80:752–758 25105392

[5] Shen X, Zhuo ZG, Li G, Alai GH, Song TN, Xu ZJ et al (2021) Is the routine placement of a feeding jejunostomy during esophagectomy worthwhile?-a systematic review and meta-analysis. Ann Palliat Med 10:4232–4241. 10.21037/apm-20-2519 33894727 10.21037/apm-20-2519

[6] Phillips AW, Dent B, Navidi M, Immanuel A, Griffin SM (2018) Trainee involvement in Ivor Lewis esophagectomy does not negatively impact outcomes. Ann Surg 267:94–98. 10.1097/SLA.0000000000002047 27759620 10.1097/SLA.0000000000002047

[7] Nakai T, Kitadani J, Ojima T, Hayata K, Katsuda M, Goda T et al (2022) Feeding jejunostomy following esophagectomy May increase the occurrence of postoperative small bowel obstruction. Med (Baltim) 101:e30746. 10.1097/MD.0000000000030746 10.1097/MD.0000000000030746 PMC9478262 36123872

[8] Koterazawa Y, Oshikiri T, Hasegawa H, Yamamoto M, Kanaji S, Yamashita K et al (2020) Routine placement of feeding jejunostomy tube during esophagectomy increases postoperative complications and does not improve postoperative malnutrition. Dis Esophagus 33. 10.1093/dote/doz021 10.1093/dote/doz021 30997494

[9] Kim MS, Shin S, Kim HK, Choi YS, Zo JI, Shim YM, Cho JH (2022) Role of intraoperative feeding jejunostomy in esophageal cancer surgery. J Cardiothorac Surg 17:191. 10.1186/s13019-022-01944-1 35987831 10.1186/s13019-022-01944-1 PMC9392926

[10] Omori A, Tsunoda S, Nishigori T, Hisamori S, Hoshino N, Ikeda A, Obama K (2022) Clinical benefits of routine feeding jejunostomy tube placement in patients undergoing esophagectomy. J Gastrointest Surg 26:733–741. 10.1007/s11605-022-05265-5 35141836 10.1007/s11605-022-05265-5

[11] Lee Y, Lu JY, Malhan R, Shargall Y, Finley C, Hanna W, Agzarian J (2022) Effect of routine jejunostomy tube insertion in esophagectomy: A systematic review and meta-analysis. J Thorac Cardiovasc Surg 164:422–32e17. 10.1016/j.jtcvs.2021.12.050 35307215 10.1016/j.jtcvs.2021.12.050

[12] Al-Temimi MH, Dyurgerova AM, Kidon M, Johna S (2019) Feeding jejunostomy tube placed during esophagectomy: is there an effect on postoperative outcomes? Perm J 23. 10.7812/TPP/18.210 10.7812/TPP/18.210 PMC6730960 31496496

[13] Alvarez-Sarrado E, Mingol Navarro F, R JR, Ballester Pla N, Vaque Urbaneja FJ, Muniesa Gallardo C et al (2019) Feeding jejunostomy after esophagectomy cannot be routinely recommended. Analysis of nutritional benefits and catheter-related complications. Am J Surg 217:114–120. 10.1016/j.amjsurg.2018.08.027 30309617 10.1016/j.amjsurg.2018.08.027

[14] Weijs TJ, van Eden HWJ, Ruurda JP, Luyer MDP, Steenhagen E, Nieuwenhuijzen GAP, van Hillegersberg R (2017) Routine jejunostomy tube feeding following esophagectomy. J Thorac Dis 9:S851–S60. 10.21037/jtd.2017.06.73 28815083 10.21037/jtd.2017.06.73 PMC5538975

[15] Kubo Y, Miyata H, Sugimura K, Shinno N, Asukai K, Hasegawa S et al (2021) Prognostic implication of postoperative weight loss after esophagectomy for esophageal squamous cell cancer. Ann Surg Oncol 28:184–193. 10.1245/s10434-020-08762-6 32591956 10.1245/s10434-020-08762-6

[16] Khaw RA, Nevins EJ, Phillips AW (2022) Incidence, diagnosis and management of malabsorption following oesophagectomy: a systematic review. J Gastrointest Surg 26:1781–1790. 10.1007/s11605-022-05323-y 35484473 10.1007/s11605-022-05323-y

[17] Lidoriki I, Schizas D, Mylonas KS, Vergadis C, Karydakis L, Alexandrou A et al (2022) Postoperative changes in nutritional and functional status of gastroesophageal cancer patients. J Am Nutr Assoc 41:301–309. 10.1080/07315724.2021.1880986 33704025 10.1080/07315724.2021.1880986

[18] Kamarajah SK, Markar SR, Phillips AW, Kunene V, Fackrell D, Salti GI et al (2022) Survival benefit of adjuvant chemotherapy following neoadjuvant therapy and oesophagectomy in oesophageal adenocarcinoma. Eur J Surg Oncol 48:1980–1987. 10.1016/j.ejso.2022.05.014 35718676 10.1016/j.ejso.2022.05.014

[19] Griffin SM, Jones R, Kamarajah SK, Navidi M, Wahed S, Immanuel A et al (2021) Evolution of esophagectomy for cancer over 30 years: changes in Presentation, management and outcomes. Ann Surg Oncol 28:3011–3022. 10.1245/s10434-020-09200-3 33073345 10.1245/s10434-020-09200-3 PMC8119401

[20] Kamarajah SK, Madhavan A, Chmelo J, Navidi M, Wahed S, Immanuel A et al (2021) Impact of smoking status on perioperative Morbidity, Mortality, and Long-Term survival following transthoracic esophagectomy for esophageal cancer. Ann Surg Oncol 28:4905–4915. 10.1245/s10434-021-09720-6 33660129 10.1245/s10434-021-09720-6 PMC8349321

[21] Jia S, Chen Y, Cui J, Wang T, Lin CC (2023) Relationship of weight loss to quality of life and symptom distress among postsurgical survivors of oesophageal cancer who received chemotherapy. Eur J Oncol Nurs 66:102370. 10.1016/j.ejon.2023.102370 37490815 10.1016/j.ejon.2023.102370

